# Supplementary material for: Development and initial validation of the Japanese healthy work environment assessment tool for critical care settings
Source: PLoS One. 2022 May 12;17(5):e0268124. doi: 10.1371/journal.pone.0268124 (PMC9098038; doi:10.1371/journal.pone.0268124)
Supplement: S1 Table — (DOCX) [file pone.0268124.s001.docx]

**S1 Table**

**Japanese version Healthy Work Environment Assessment Tool**

| Q1 | 看護部管理者、看護師長、医師、看護師、その他のスタッフは、お互  いに決定事項に驚いたり、不意をつかれる思いをしたりしないように、  頻繁なコミュケーションを保っている。 |
| --- | --- |
| Q2 | 看護部管理者、看護師長、医師は重要な決定を下す際、然るべき程度に看護師やその他のスタッフを関与させている。 |
| Q3 | 看護部管理者や看護師長は、看護師やその他のスタッフと協働し、患者の安全を維持するために十分な人材を確保するよう努めている。 |
| Q4 | 看護師やその他のスタッフが自らの価値を実感できるよう、公式な報奨と表彰の仕組みが機能している。 |
| Q5 | 大多数の看護師とその他のスタッフは、リーダーシップをとる看護師たち（看護師長、主任看護師、認定看護師、専門看護師など）と良好な人間関係を構築できている。 |
| Q6 | 看護部管理者、看護師長、医師、看護師、その他のスタッフは、言動が一貫するように努めており、「言行一致」されている。 |
| Q7 | 看護部管理者、看護師長、医師、看護師、その他のスタッフは、自身らの決定が最も高い質を保てるように、一貫してデータを用いた論理的な意思決定プロセスを踏んでいる。 |
| Q8 | 看護部管理者と看護師長は、最適な結果が得られるよう看護師と他のスタッフが職場内に適切に混在するように努めている。 |
| Q9 | 看護部管理者、看護師長、医者、看護師、そしてその他のスタッフメンバーは、誰かが良い仕事をやり遂げたときには、称賛し、みんなに知らせている。 |
| Q10 | 看護師とその他のスタッフは、病院の方針や手順、官僚的な組織体制に対し、影響を及ぼすことができると感じている。 |
| Q11 | 重要な決定を下すときには、適切な部署、専門職、グループが関わっている。 |
| Q12 | 患者とその家族のケアに関する優先順位の高いものや必須事項に時間をかけられるように、看護師とその他のスタッフへのサポートが提供されている。 |
| Q13 | リーダーシップをとる看護師たち（看護師長、主任看護師、認定看護師、専門看護師など）は、ケアに求められていることと、ケアで生じるチームダイナミクスを理解していることを行動で示し、この知識をより良い仕事環境作りに活かしている。 |
| Q14 | 看護部管理者、看護師長、医師、看護師、その他のスタッフは、いかな  る場合であっても相手を尊重しない態度や不適切な扱いを認めない。も  し、無礼な態度を見聞きした場合は、個人の役割や職位に関わらず責任  を課す。 |
| Q15 | 看護部管理者、看護師長、医師は看護師や他のスタッフと話をするとき、一方的なコミュニケーションや指示を与えるのではない。むしろ、彼らから意見を求めて意思決定に活用する。 |
| Q16 | 看護部管理者、看護師長、医者、看護師、その他のスタッフは、重要な決定を下す際は常に、患者や家族の視点に立って考慮することに気をつけている。 |
| Q17 | 自己成長、自己啓発、昇進のための意欲を引き出す機会がある。 |
| Q18 | リーダーシップをとる看護師たち(看護師長、主任看護師、認定看護師、専門看護師など)は、非常に重要な決定を行う過程の中で、役割を果たすための参加と必要な権限が与えられている。 |
