# Supplementary material for: Development and initial validation of the Japanese healthy work environment assessment tool for critical care settings
Source: PLoS One. 2022 May 12;17(5):e0268124. doi: 10.1371/journal.pone.0268124 (PMC9098038; doi:10.1371/journal.pone.0268124)
Supplement: S2 Table — (DOCX) [file pone.0268124.s002.docx]

**S 2 Table**

**Measures of Sampling Adequacy**

| Overall Measures of Sampling Adequacy | 0.93 |
| --- | --- |
| Measures of Sampling Adequacy for each item | |
| Q1 | 0.87 |
| Q2 | 0.94 |
| Q3 | 0.93 |
| Q4 | 0.94 |
| Q5 | 0.89 |
| Q6 | 0.94 |
| Q7 | 0.93 |
| Q8 | 0.95 |
| Q9 | 0.92 |
| Q10 | 0.91 |
| Q11 | 0.95 |
| Q12 | 0.769 |
| Q13 | 0.71 |
| Q14 | 0.82 |
| Q15 | 0.93 |
| Q16 | 0.90 |
| Q17 | 0.95 |
| Q18 | 0.94 |
