# Supplementary material for: Development and initial validation of the Japanese healthy work environment assessment tool for critical care settings
Source: PLoS One. 2022 May 12;17(5):e0268124. doi: 10.1371/journal.pone.0268124 (PMC9098038; doi:10.1371/journal.pone.0268124)
Supplement: Attachment — Submitted filename: Review comments.docx [file pone.0268124.s005.docx]

Thank you for asking me to review this article. The results are very interesting, the article is clearly written and organized, and I believe the article will be of interest to the readers.

There are some minor fixes to consider.

**Introduction**

On page 5, line 89, a few more recent studies examining the impact of the work environment on patient and employee health/outcomes should be included.

The Covid-19 pandemic has been an important factor affecting intensive care environments, workforce planning, rapid decision making, communication and collaboration. This is not mentioned at all in the article. Briefly, the effect of the pandemic on the intensive care work environment can be mentioned.

**Background**

Page 6, line 99. Does this measurement tool (HWE-AT) have a cutoff score? How is the score obtained from the scale interpreted? It should be explained briefly.

**Methods/ Content validity**

What technique was content validation done (Davis?)

Page 8, line 141. It is the expert opinion experienced in the hospital setting? Or were those with intensive care experience selected?

**Participant Selection for Construct Validity**

Is the delivery of care in intensive care units in Japan cascaded according to the severity of the patient condition? Which level (1st, 2nd or 3rd level) of the nurses participating in the study work in the intensive care unit?

**Results**

**Pilot testing**

Were 40 nurses participating in the study included in the sample of the study at this stage? It must be disclosed.

**Sample size**

Page 10, line 184. Has the sample size been confirmed by the Kaiser-Meyer-Olkin test as to whether the sample size is sufficient for factor analysis?

Before the discussion, the characteristics of the final form should be given briefly. It is as if any item was removed from the Japanese scale and the sub-factor structure was preserved.

**Clinical Implications/Research Implication**

Page 17, lines 301-302. “standard” should be explained, whether a unit falls below the standard will be evaluated according to a written/valid procedure or according to the score obtained from the scale!

**Figure 1**

“Testeing” should be fixed
